# Cathepsin K Deficiency Prevents the Aggravated Vascular Remodeling Response to Flow Cessation in ApoE^-/-^ Mice

**DOI:** 10.1371/journal.pone.0162595

**Published:** 2016-09-16

**Authors:** Marjo M. P. C. Donners, Lili Bai, Suzanne P. M. Lutgens, Erwin Wijnands, Jason Johnson, Leon J. Schurgers, Cong-Lin Liu, Mat J. A. P. Daemen, Kitty B. J. M. Cleutjens, Guo-Ping Shi, Erik A. L. Biessen, Sylvia Heeneman

**Affiliations:** 1 Experimental Vascular Pathology group, Department of Pathology, Cardiovascular Research Institute Maastricht (CARIM), Maastricht University Medical Center, Maastricht, The Netherlands; 2 Department of Biochemistry, Cardiovascular Research Institute Maastricht (CARIM), Maastricht University Medical Center, Maastricht, The Netherlands; 3 Department of Pathology, Amsterdam Medical Center, Amsterdam, The Netherlands; 4 Bristol Heart Institute, University of Bristol, Bristol Royal Infirmary, Marlbrough Street, Bristol, BS2 8HW, United Kingdom; 5 Cardiovascular Medicine, Brigham and Women's Hospital, NRB-742, 77 Avenue Louis Pasteur, Boston, Massachusetts, 02115, United States of America; Uniwersytet Gdanski, POLAND

## Abstract

Cathepsin K (catK) is a potent lysosomal cysteine protease involved in extracellular matrix (ECM) degradation and inflammatory remodeling responses. Here we have investigated the contribution of catK deficiency on carotid arterial remodeling in response to flow cessation in apoE^-/-^ and wild type (wt) background. Ligation-induced hyperplasia is considerably aggravated in apoE^-/-^ versus wt mice. CatK protein expression was significantly increased in neointimal lesions of apoE^-/-^ compared with wt mice, suggesting a role for catK in intimal hyperplasia under hyperlipidemic conditions. Surprisingly, CatK deficiency completely blunted the augmented hyperplastic response to flow cessation in apoE^-/-^, whereas vascular remodeling in wt mice was unaffected. As catK deficiency did neither alter lesion collagen content and elastic laminae fragmentation in vivo, we focused on effects of catK on (systemic) inflammatory responses. CatK deficiency significantly reduced circulating CD3 T-cell numbers, but increased the regulatory T cell subset in apoE^-/-^ but not wt mice. Moreover, catK deficiency changed CD11b+Ly6G-Ly6C ^high^ monocyte distribution in apoE^-/-^ but not wt mice and tended to favour macrophage M2a polarization. In conclusion, catK deficiency almost completely blunted the increased vascular remodeling response of apoE^-/-^ mice to flow cessation, possibly by correcting hyperlipidemia-associated pro-inflammatory effects on the peripheral immune response.

## Introduction

CatK is the most potent mammalian elastase of the cathepsin family of proteases yet known and possesses collagenolytic as well as elastinolytic activity [1, 2]. Next to its involvement in extracellular matrix (ECM) degradation, catK was reported to modulate inflammatory responses via the TGF-β [3] and TLR-9 [4] signaling pathway. In a previous study, apoE^-/-^ mice with a targeted disruption of the catK gene showed reduced atherosclerotic lesion growth [5]. In addition, uptake of modified lipoproteins was enhanced in catK^-/-^ macrophages, potentially via a caveolin-1- and scavenger receptor CD-36- dependent pathway [3, 5]. Selective disruption of leukocyte catK was seen to dramatically decrease collagen and increase macrophage content of atherosclerotic lesions, leaving lesion size unaffected. It was suggested that smooth muscle cell (SMC) migration was hampered due to the abolished elastinolytic activity of macrophage catK[5]. Clearly both these studies suggested a role for macrophage catK in atherosclerosis.

In the vascular remodeling process, proliferation and migration of SMC from the media to the intima contribute to neointima formation [6, 7]. Migration of SMCs requires degradation of ECM. Indeed intimal mRNA and protein levels of catK were found to be enhanced after balloon-injury of rat carotid arteries [8]. There was a significant increase in elastolytic and collagenolytic activity in the intima tissue extracts compared with uninjured control vessels [8]. The augmented ECM degrading potential and expression and activity of catK during intimal hyperplasia suggests a role for catK in vascular remodeling. To further study the potential role of catK in vascular remodeling, we compared carotid artery remodeling and neointima formation in a flow cessation model in both catK-deficient apoE^-/-^ and catK-deficient wild type (wt) backgrounds. In this model, flow cessation is used to accelerate the formation of macrophage-rich neointima lesions in hypercholesteremic apoE-deficient mice and the formation of a macrophage-poor neointima lesion in the wt mice, respectively [9]. CatK deficiency led to attenuated macrophage-rich neointima formation in the hyperlipidemic apoE^-/-^ mice, while macrophage-poor neointima formation in C57Bl6/wt mice was unaffected.

## Materials and Methods

### Experimental model of flow cessation induced intimal hyperplasia/vessel retraction

Male apoE^-/-^ mice on a C57Bl6/J background were obtained from IffaCredo (Lyon, France). CatK^-/-^ mice, kindly provided by Dr P Saftig, were generated on an outbred 129SVJ-C57BL/6J genetic background [10]. We subsequently backcrossed the catK^-/-^ mice at least 9 times with C57BL6/J mice and apoE^-/-^ mice to generate catK^+/+^ and catK^-/-^ mice on a C57BL6/J (wt) or apoE^-/-^ background, respectively. Animals were maintained in accordance with the Dutch government guidelines and animal experiments were approved by the regulatory authority of Maastricht University (DierExperimenten Commissie (DEC)).

Macrophage-rich and -poor intimal lesions were induced by unilateral flow cessation of the common carotid artery in hypercholesterolemic apoE^-/-^ and wt mice, respectively as described by Ivan et al [9]. In brief, mice were anesthetized with 2.5% isofluorane, and blood flow in the common right carotid artery was disrupted by ligation with a silk suture (5–0) near the bifurcation. Wt and catK^-/-^ mice from C57Bl6/J background (17–18 weeks of age) were fed a normal chow (SNIFF, V1534) throughout the experiment, while apoE^-/-^ and catK^-/-^//apoE^-/- ^mice (13–16 weeks of age) were fed a western type diet containing 0.25% cholesterol (Research Diet, diet number 4021.06) from two weeks before ligation onwards [9, 11].

### Tissue harvesting and analysis

Four weeks after ligation, mice were sacrificed. The arterial tree was perfused with PBS containing 0.1 mg/ml nitroprusside (Sigma, St Louis, MO) and subsequently with 1% paraformaldehyde via a catheter inserted into the left cardiac ventricle. Right carotid artery cross-sections (4 μm thick) were cut at 100 μm intervals. At each level of 100 μm, a cross-section was stained with Lawson solution and used to determine intima, lumen and total vessel area as well as elastic lamina fragmentation. The relative intimal collagen content, i.e. the percentage of total intima area that stained positive for Sirius red, was determined under a microscope coupled to a computerized morphometry system (Quantimet 570, Leica). Morphometric analyses were performed by a blinded investigator (LB, intra-observer variability was < 10%) using a computerized morphometry system (Quantimet 570, Leica). Serum cholesterol level was determined with the CHOD-PAP method (Roche Diagnostics).

### Immunohistochemistry

Immunohistochemical stainings were performed to determine macrophage (anti-MAC3), T cell (anti-CD3+) and SMC (α-smooth muscle actin, α-SMA)) content, as described previously [5]. The relative intimal macrophage, T cell and SMC content, was calculated by dividing the number of lesional MAC3+, CD3+ and α-SMA+ cells, respectively by the total number of cells in the lesion. Expression of catK was determined using a goat anti-human catK (c16) antibody (1:100, Santa Cruz, USA). The relative catK-positive area was determined by a computerized morphometry (Quantimet 570, Leica). Mouse spleen was used as positive control for CD3 and catK staining, and mouse atherosclerotic lesions as positive control of both MAC3 and α-SMA staining.

To localize CatK to macrophages de-paraffinized slides were treated with Heat-induced antigen Retrieval Solution at 95°C and blocked in PBS supplemented with 5% normal goat serum and 2.5% BSA for 1 hr. Sections were incubated overnight at 4°C with a mixture of rabbit anti-CatK (1:100, CalBiochem) and rat anti-mouse Mac 2 (1:100, Cedarlane) in PBS supplemented with 5% Normal Goat Serum + 2.5% BSA. After washing in PBS, a mixture of secondary antibodies: goat anti- rabbit AlexaFluor 555 (1:500, Life Tech, Invitrogen) and donkey anti-rat AlexaFluor 488 (1:300, Lifesciences) was applied for 45 minutes at RT.

To localize CatK to SMC, after the same pre-treatment with HRS and block, sections were incubated overnight with Cat K (1:100) followed by goat anti- rabbit AlexaFluor 555 (1:500). After washing in PBS, FITC-conjugated mouse anti-α actin (1:500, Sigma) antibody was applied for 1 hour at RT. Slides were counterstained with DAPI (Molecular Probes) and coverslipped (Dako Fluorescent Mount Medium). Image acquisition was performed using FluoView 1000 confocal laser scanning microscope (Olympus, Tokyo, Japan).

Deletion of the primary antibody served as negative control. No positive staining for any of the antibodies was observed on negative controls. All measurements were conducted by a single investigator (LB, intra-observer variability was < 10%).

### Fluorescence-activated cell sorting (FACS)-analysis

Splenocytes and blood were isolated from apoE^-/-^, catK^-/-^//apoE^-/-^, wt, and catK^-/-^ mice. Erythrocytes in peripheral blood and spleen were removed by hypotonic lysis with NH_4_Cl. Cells were incubated first with anti-CD16/32 (eBioscience, San Diego, CA) to block Fc receptor binding to antibodies on macrophages, neutrophils and mast cells and stained with anti-CD3-FITC, anti-CD8-Pacific blue, anti-CD25-APC, anti-CD45R(B220)-Pe-Cy7 (eBioscience, San Diego, CA) and anti-CD4-PerCp (BD-Biosciences Pharmingen, San Diego, CA). Foxp3-positive cells were detected with PE anti-mouse/rat Foxp3 Staining Set, according to the manufacturer’s instruction (eBioscience, USA). Peripheral blood leukocytes were incubated with anti-CD11b-pacific blue (eBioscience, San Diego, CA) and anti-ly6G-PE (BD-Biosciences Pharmingen, San Diego, CA) to detect monocytes (CD11b^+^ly6G^-^) and granulocytes (CD11b^+^ly6G^+^). For measurement of inflammatory monocytes (CD11b+Ly6G-Ly6C^high^) cells were stained with anti-Ly6C-FITC (Miltenyi Biotec). Blood natural killer (NK) cells were stained by anti-CD49b(DX5)-APC (Miltenyi Biotec).

### Cell isolation and culturing

Bone marrow–derived macrophages (BMM) were isolated from the femur and tibia of wt, catK^-/-^, apoE^-/-^, and catK^-/-^ //apoE^-/-^ mice. Cells were cultured in standard RPMI culture medium containing L-glutamine, HEPES, 10% fetal calf serum, 100 IU/mL penicillin/streptomycin, and 15% L929 cell conditioned medium (containing Macrophage-Colony Stimulating Factor to drive macrophage differentiation).

Primary vascular SMC were isolated from aortas of apoE^-/-^ mice. Aortas were stripped from left-over fat, connective tissue and adventitia. Endothelial cells were removed and aorta was cut into small pieces. Aorta pieces were digested with collagenase and elastase for four hours in which they were mixed every 30 minutes. After digestion cells were seeded in T25 culture flasks coated with laminin. Cells were left to grow for at least 72 hours and were used after they reached passage 3.

### Cell proliferation assay

SMCs (2x10^4^ cells) were seeded on a gold electrode implemented in a 96 wells plate and allowed to attach for 24h. Cells were treated with 0–0.1 μM Cathepsin K inhibitor II (Calbiochem) and impedance was measured using the XCelligence system (Roche) according to manufacturer’s protocol.

### Quantitative RT-PCR

RNA was extracted from BMM lysate with Nucleospin RNA II kit (MACHEREY-NAGEL, Duren, Germany). cDNA was generated with iSCript ^™^ CDNA synthesis kit (BIO-RAD, Hercules, CA). Real-time PCR was done with a Taqman IQ ^™^ SYBR Green Super Mix (BIO-RAD, Hercules, CA). Cyclophilin A was used to normalize RNA quantity. Primer sequences of arginase-1, MR (mannose receptor), IL-10, Nos2, IL-18 and cyclophilin are shown in S1 Table. The data were expressed as ratio of the quantity of specific transcripts to the quantity of the cyclophilin A gene. The relative gene expression data from wt, catK^-/-^, apoE^-/-^ and catK^-/-^//apoE^-/-^ background was then normalized to the data of wt background.

### Statistics

Statistical analyses were performed using a nonparametric Mann–Whitney *U* test. Data are expressed as mean±SEM, and differences were considered statistically significant at *P*<0.05.

## Results

### Intimal lesions in apoE^-/-^ mice have increased catK protein expression

**Fig 1:**
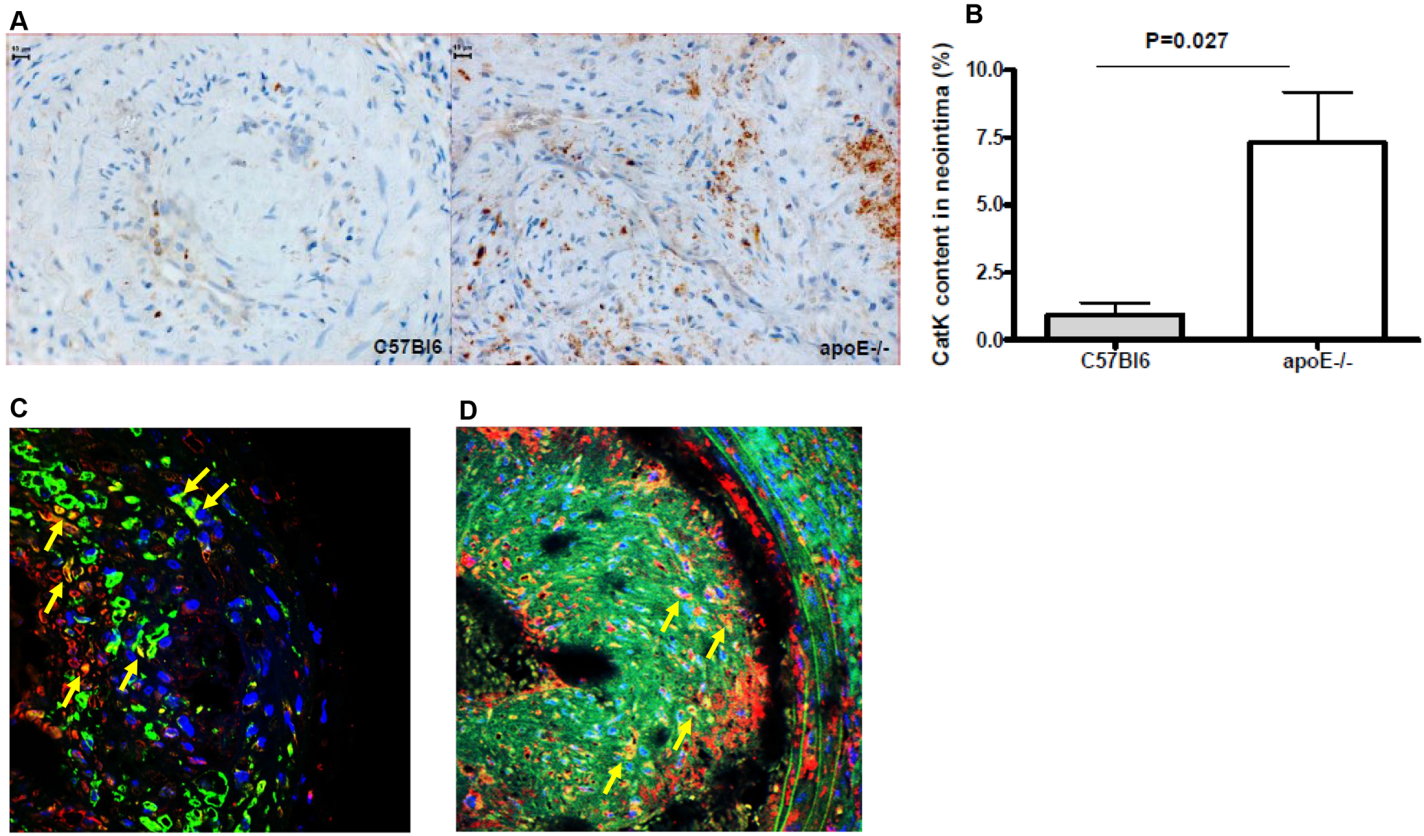
CatK expression in macrophage-rich (apoE^-/-^) and macrophage-poor (wt) flow cessation-induced intimal lesions. A. Representative pictures of catK staining in wt and apoE^-/-^ mice. B. Relative catK-positivity was calculated by dividing the anti-catK Ab stained area by the total intima area (P = 0.027; n = 5 for wt, n = 12 for apoE^-/-^). C-D. Representative examples of CatK (red) double staining with Mac2+ macrophages (left panel, green) or α-actin+ SMCs (right panel, green). DAPI (Blue) was used to stain cell nuclei. Arrows indicate double-positive cells.

To study the role of catK in macrophage-poor and macrophage-rich neointima formation, we first examined catK protein expression in such lesions. CatK expression in flow cessation-induced intimal lesions of wt mice was very low and mainly confined to SMCs and the ECM (n = 5). In contrast, macrophage-rich lesions of apoE^-/-^ mice showed pronounced expression of catK (n = 12, Fig 1), primarily residing in cytoplasm of macrophages (Fig 1C), but also in SMCs (Fig 1D) and, in a more diffuse manner, in the ECM (Fig 1A).

### CatK deficiency attenuates flow cessation-induced intimal hyperplasia in apoE^-/-^ but not wt mice

Next we examined the formation of macrophage-poor and macrophage-rich neointima in catK deficient mice on a wt or apoE^-/-^ background, respectively. In line with our previous atherosclerosis studies, catK deficiency did not alter serum cholesterol level in neither apoE^-/-^ nor wt mice (n = 14–15 for apoE^-/-^ and catK^-/-^//apoE^-/-^ mice; n = 7 for wt and catK^-/-^ mice, S1 Fig).

**Fig 2:**
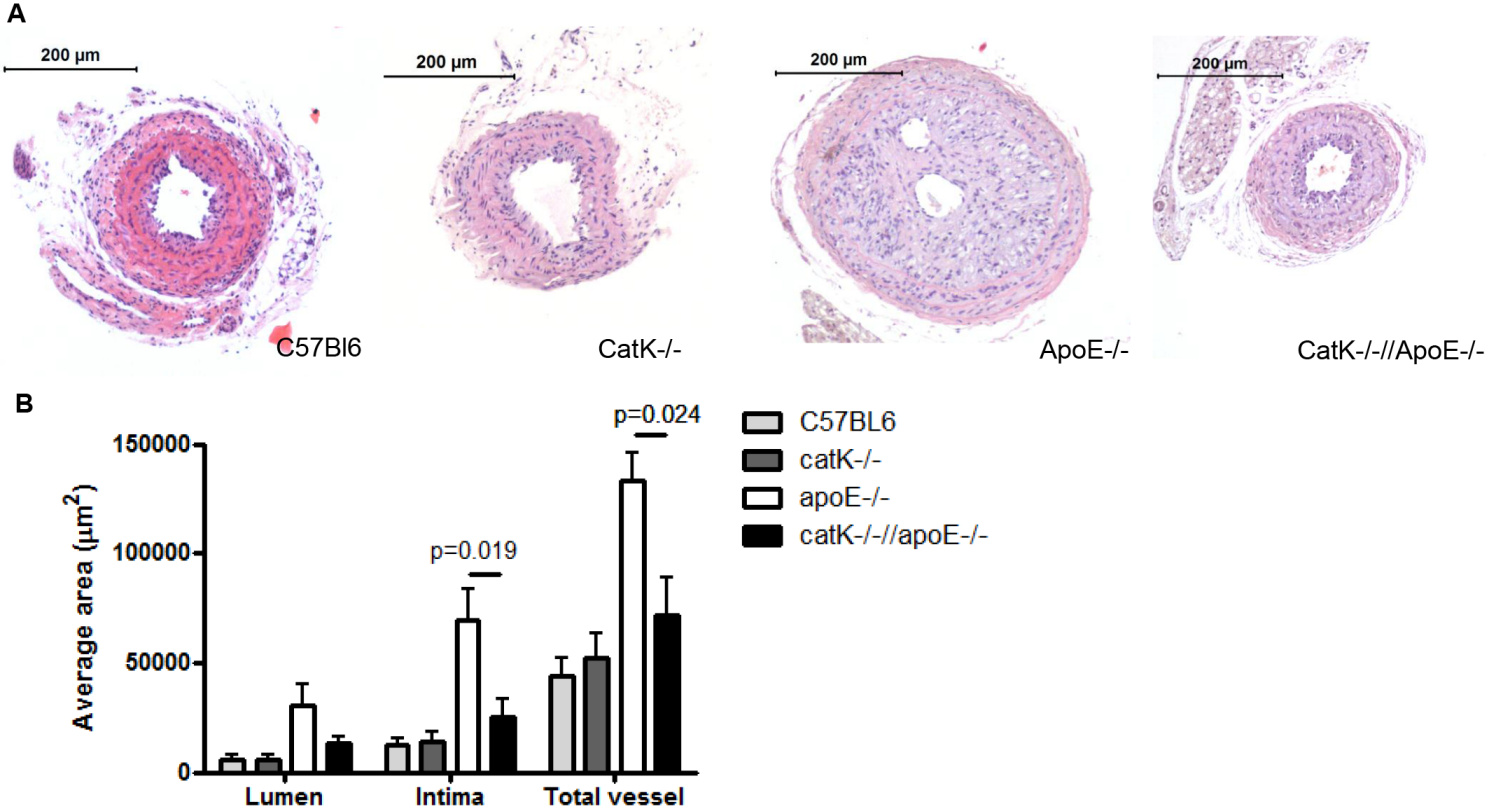
Effect of catK deficiency on intimal hyperplasia. A. Representative micrographs showing HE staining of intima lesions in wt, catK^-/-^, apoE^-/-^ and catK^-/-^//apoE^-/-^ mice. B. CatK deficiency did not affect lumen, intima and total vessel area in wt mice. CatK deficiency significantly reduced intima (P = 0.019) and total vessel area (P = 0.024) in apoE^-/-^ mice. Values represent the mean ± SEM. (n = 6–8 for wt and catK^-/-^ mice, n = 14–15 for apoE^-/-^ and catK^-/-^//apoE^-/-^ mice).

ApoE deficiency significantly enhanced lumen area (p = 0.027), average lesion area (p = 0.006) and total vessel area (p = 0.009) compared to wt background, indicating a prominent outward remodeling in apoE^-/-^ background. Average lesion area, total vessel and lumen area were comparable in wt and catK^-/-^ mice on chow diet (n = 6–8, Fig 2 –gray bars). In sharp contrast, catK deficiency was found to prevent intimal hyperplasia in apoE^-/-^ mice. Average lesion and total vessel area were significantly reduced in catK^-/-^//apoE^-/-^ compared to apoE^-/-^ mice, whereas lumen area was not significantly different (n = 14–15, Fig 2 –black and white bars). In fact, lesion size and total vessel area of catK^-/-^//apoE^-/-^ mice were almost identical to that of normolipidemic wt and catK^-/-^ mice. Thus, apoE deficiency induced a prominent outward remodeling and neointimal growth in ligated right carotid artery compared to wt, which was almost completely reversed by CatK deficiency.

### CatK deficiency did not alter lesion composition in apoE^-/-^ and wt mice

**Fig 3:**
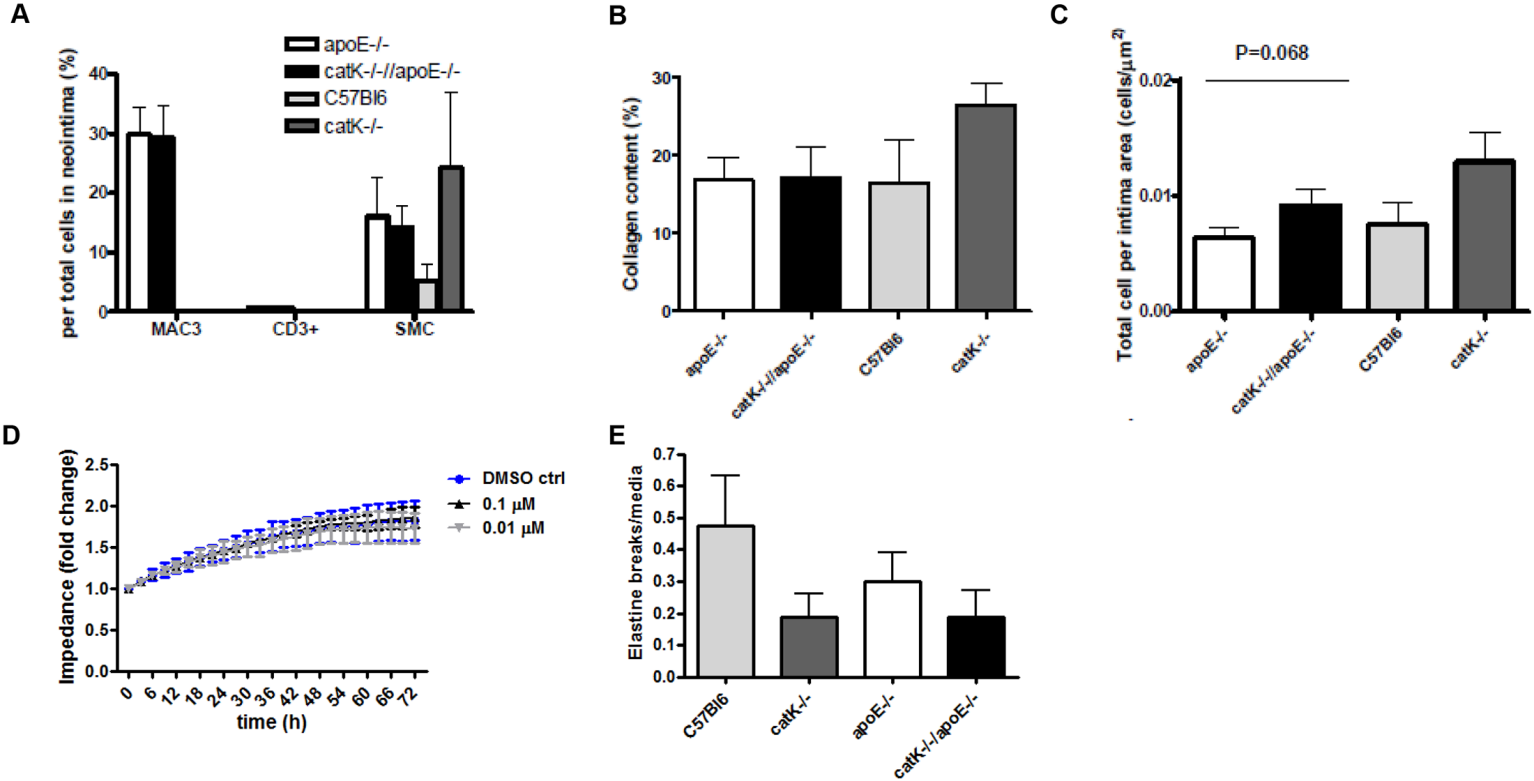
CatK deficiency did not affect neointimal lesion composition. (A) Macrophage, CD3 and SMC content in the macrophage-rich lesions of apoE^-/-^ mice or in macrophage-poor lesions of wt mice was not altered by CatK deficiency. The number of MAC3-positive, CD3-positive and α-SMA-positive cells in the intima area were corrected for the total number of cells in the entire intima area. CatK deficiency did not change collagen content in the intima area in apoE^-/-^ and wt mice (B). CatK deficiency did not significantly alter cell density in the intima area in apoE^-/-^ and wt mice (C). Total cell number in neointima lesion was corrected for the corresponding lesion area. Values represent the mean ± SEM. (n = 6–8 for wt and catK^-/-^ mice, n = 14–15 for apoE^-/-^ and catK^-/-^//apoE^-/-^ mice). D: CatK inhibition did not affect *in vitro* primary mouse SMC proliferation. E: CatK deficiency did not affect medial elastin break frequency in apoE^-/-^ and in WT mice. Frequency is expressed as average number of breaks per cross-section.

SMC, collagen content and total cell density of macrophage-poor lesions of WT and catK^-/-^ mice were comparable (n = 6–8, Fig 3A–3C). There was no difference in total cell density, SMC content and collagen content between lesions of wt and apoE^-/-^ mice, although as expected lesions in apoE^-/-^ but not WT were enriched in macrophages (Fig 3A–3C, S2 Fig). Macrophage number (n = 14–15, Fig 3A), CD3^+^ T cell number (n = 14–15, Fig 3A) SMC number (n = 13–15, Fig 3A) content as well as collagen content (n = 14–15, Fig 3B) did not differ between apoE^-/-^//catK^-/-^ and apoE^-/-^ mice. Total cell number tended to be higher in macrophage-rich lesions of catK^-/-^//apoE^-/-^ than of apoE^-/-^ mice (n = 14–15, Fig 3C). *In vitro*, we could rule out any effects of CatK on SMC proliferation (Fig 3D). As cleaved caspase-3 positive cells were scant but present at essentially similar numbers in catK^-/-^//apoE^-/-^ and apoE^-/-^ lesions (data not show), the increased cell density in catK^-/-^//apoE^-/-^ mice is probably not attributable to a decrease in apoptotic rate.

CatK deficiency did not affect medial elastic laminae fragmentation in both mouse strains (n = 6–8 for wt background and 14 for apoE^-/-^ background, respectively; Fig 3E).

### Peripheral immune activity was altered in catK deficient apoE^-/-^ mice but not in catK deficient wt mice

**Fig 4:**
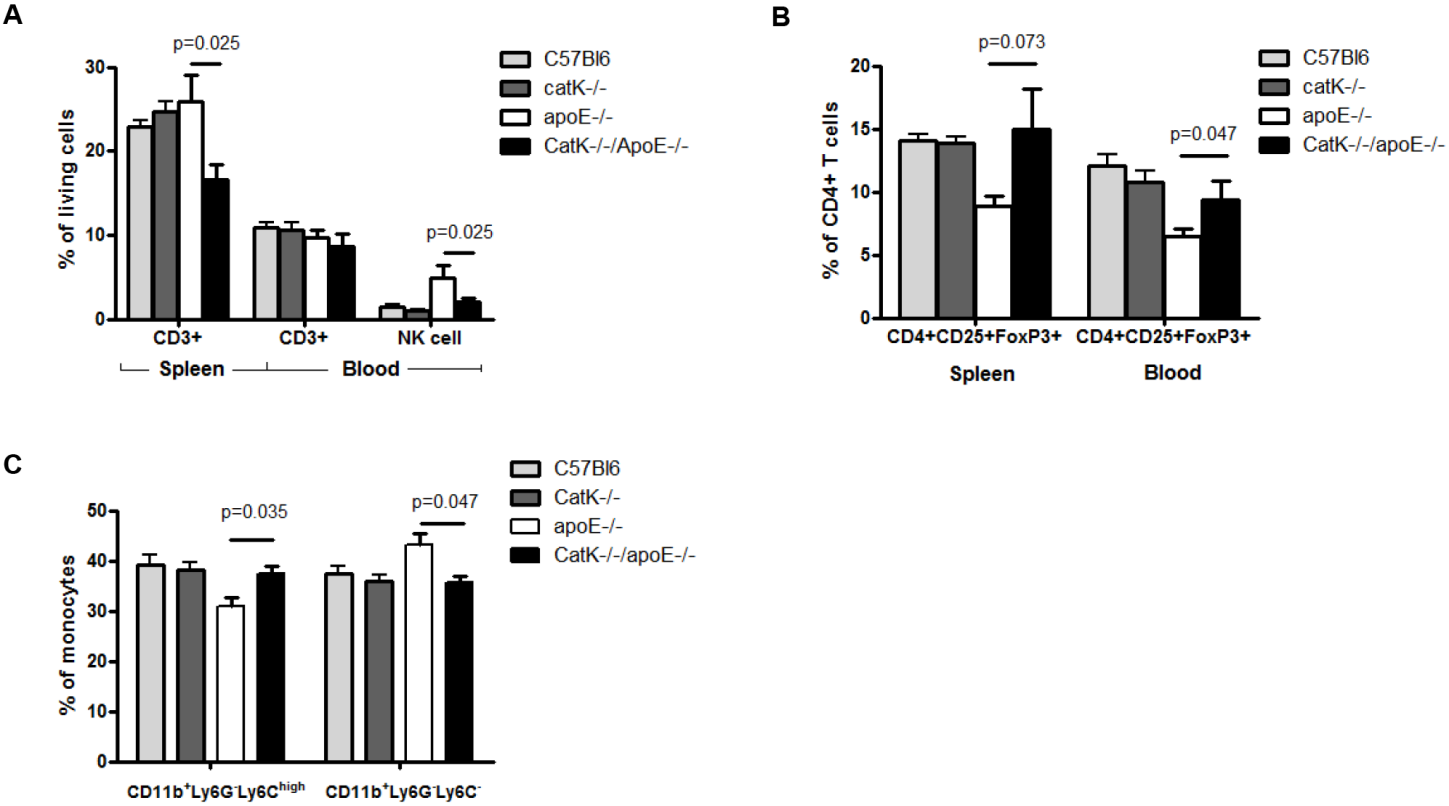
Flow-cytometric analysis of the effect of catK deficiency on CD3^+^, B, NK, CD4+CD25+Foxp3 regulatory T cells and CD11b+Ly6G-Ly6C^high/-^ monocytes in spleen and blood. Percentage of CD3^+^ T cells in spleen (P = 0.025) and NK cells in blood (P = 0.025) were significantly reduced in catK^-/-^//apoE^-/-^ mice compared with apoE^-/-^ (A). The regulatory T cell population defined as CD4+CD25+Foxp3+ cells, was significantly increased in catK^-/-^//apoE^-/-^ mice in blood (B, P = 0.025). CD11b+Ly6G-Ly6C^high^ monocytes were significantly increased (P = 0.035) and CD11b+Ly6G-Ly6C^-^ monocytes significantly reduced (P = 0.047) in blood of catK^-/-^//apoE^-/-^ compared with apoE^-/-^ mice (C). CatK deficiency in the wt mice did not affect peripheral immune activity (D, E and F). Values represent the mean ± SEM. (n = 6 for each group).

Our data indicate that catK deficiency only impacts outward remodeling in apoE^-/-^ mice, the lesions of which are enriched in leukocytes. As intimal leukocytes have been implicated in expansive arterial remodeling [9], we investigated leukocyte populations and the inflammatory status of the mice in more detail. First, we mapped peripheral immune cell profiles in normo- (wt) and hyperlipidemic (apoE^-/-^) mice by flow cytometry. Although there was no difference in CD3+ T cells (expressed as percentage of total living cells) in spleen and blood between wt and apoE^-/-^ mice, natural killer (NK) cells were significantly increased in blood of apoE^-/-^ compared with wt mice (p = 0.0041, Fig 4A). The percentage of regulatory T cells (CD4+CD25+FoxP3+ relative to CD4+ T cells) in spleen (p = 0.0021) and blood (p = 0.0012) on the other hand were dramatically reduced in apoE^-/-^ compared with wt mice (Fig 4B). In addition, apoE^-/-^ mice showed significantly decreased levels of inflammatory monocytes (CD11b+Ly6G-Ly6C ^high^) in blood compared with wt mice (Fig 4C). Thus apoE^-/-^ mice had elevated NK cell but decreased regulatory T cell and inflammatory Ly6C^high^ monocyte numbers compared with wt mice.

CatK deficiency in normolipidemic C57Bl6 mice, did not affect these leukocyte populations. However in apoE^-/-^ mice, CatK deficiency increased levels of inflammatory monocytes (CD11b+Ly6G-Ly6C ^high^) in blood, while patrolling monocytes (CD11b+Ly6G-Ly6C ^low^) were reduced (n = 6, Fig 4C). Furthermore, CD3+ T cells in spleen as well as NK cells in blood were reduced (n = 6, Fig 4A), whereas the percentage of regulatory T cells (CD4+CD25+FoxP3+ relative to CD4+ T cells) was significantly increased in both blood and spleen of catK^-/-^//apoE^-/-^ mice (n = 6, Fig 4B). Thus, catK deficiency normalizes inflammatory monocyte and regulatory T cell levels in apoE^-/-^ mice to levels found in wt.

All together these data indicate that the systemic anti-inflammatory potential of catK deficiency in apoE^-/-^ mice might lead to a protection against macrophage-rich lesion formation after carotid artery ligation that has been characterized by stronger inflammatory responses compared to the macrophage-poor lesions in normolipidemic mice [12].

### CatK deficiency promotes polarization towards a ‘wound healing’ macrophage phenotype

Several studies have shown that macrophage-subset derived inflammatory mediators and growth factors contribute to intimal lesion enlargement [13–15]. CatK deficiency has been shown to affect macrophage foam cell formation [5], however the effect of catK on macrophage polarization is unknown. We therefore studied the effect of catK deficiency on macrophage polarization in vitro. The expression of M2 macrophage markers such as arginase-1 and mannose receptor (markers for M2a macrophage), IL-10 (marker for M2b macrophage), and M1 macrophage markers such as IL-18 and Nos2 were measured in BMM from catK^-/-^//apoE^-/-^, apoE^-/-^, catK^-/-^, and wt mice without prior stimulation to assess baseline polarization.

**Fig 5:**
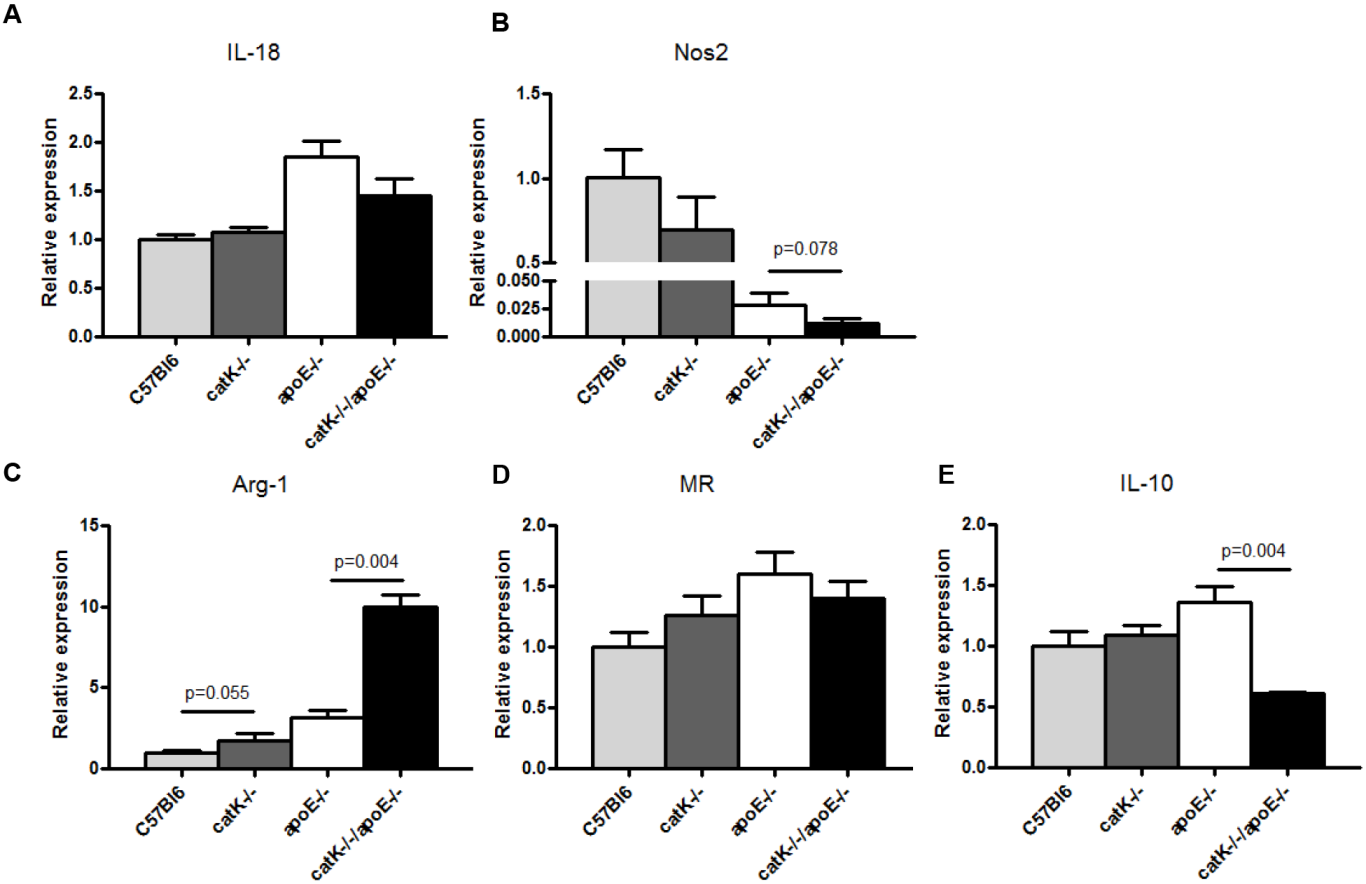
Effect of catK deficiency on M1 and M2 macrophage marker expression of non-stimulated BMM. BMM from wt, catK^-/-^, apoE^-/-^ and catK^-/-^//apoE^-/-^ mice were isolated and BMM cell lysates were analyzed for M1 and M2 marker expression. CatK^-/-^//apoE^-/-^ -derived BMM increased arginase-1 expression (C, P = 0.004) and significantly decreased IL-10 expression (E, P = 0.004) compared with apoE^-/-^ -derived-BMM. While IL-18 (A) and Mannose Receptor (D) were not affected, catK deficiency tended to decrease Nos2 expression in ApoE^-/-^ BMM (B). Values represent the mean ± SEM. (n = 6 for each group).

Compared to wt, apoE^-/-^ significantly enhanced both M1 macrophage marker IL-18 (p = 0.006, Fig 5A) and M2 macrophage markers arginase-1 (p = 0.004, Fig 5C), MR (p = 0.037, Fig 5D) and IL-10 (p = 0.055, Fig 5E). Of note, Nos2 expression was significantly reduced in apoE^-/-^ mice compared to wt (p = 0.004, Fig 5B).

CatK deficiency in wt mice did not markedly alter macrophage polarization, although catK^-/-^ arginase-1 expression tended to be enhanced (n = 6, Fig 5C). Interestingly, BMM-derived from catK^-/-^//apoE^-/-^ mice displayed a significantly decreased expression of IL-10 (n = 6, Fig 5E), while arginase-1 expression was considerably increased compared with BMM-derived from apoE^-/-^ mice (n = 6, Fig 5C). In addition, a tendency to a decreased expression of the M1 macrophage marker Nos2 was found in catK^-/-^//apoE^-/-^ BMM compared with apoE^-/-^ BMM (p = 0.078, n = 6, Fig 5B), while there was no difference in IL-18 and MR expression between catK^-/-^//apoE^-/-^ and apoE^-/-^ -derived BMM (n = 6, Fig 5A and 5D). Therefore, deficiency of catK in the apoE^-/-^ background skewed basal macrophage polarization towards an arginase-1^+^ (‘wound healing’) macrophage phenotype.

**Fig 6:**
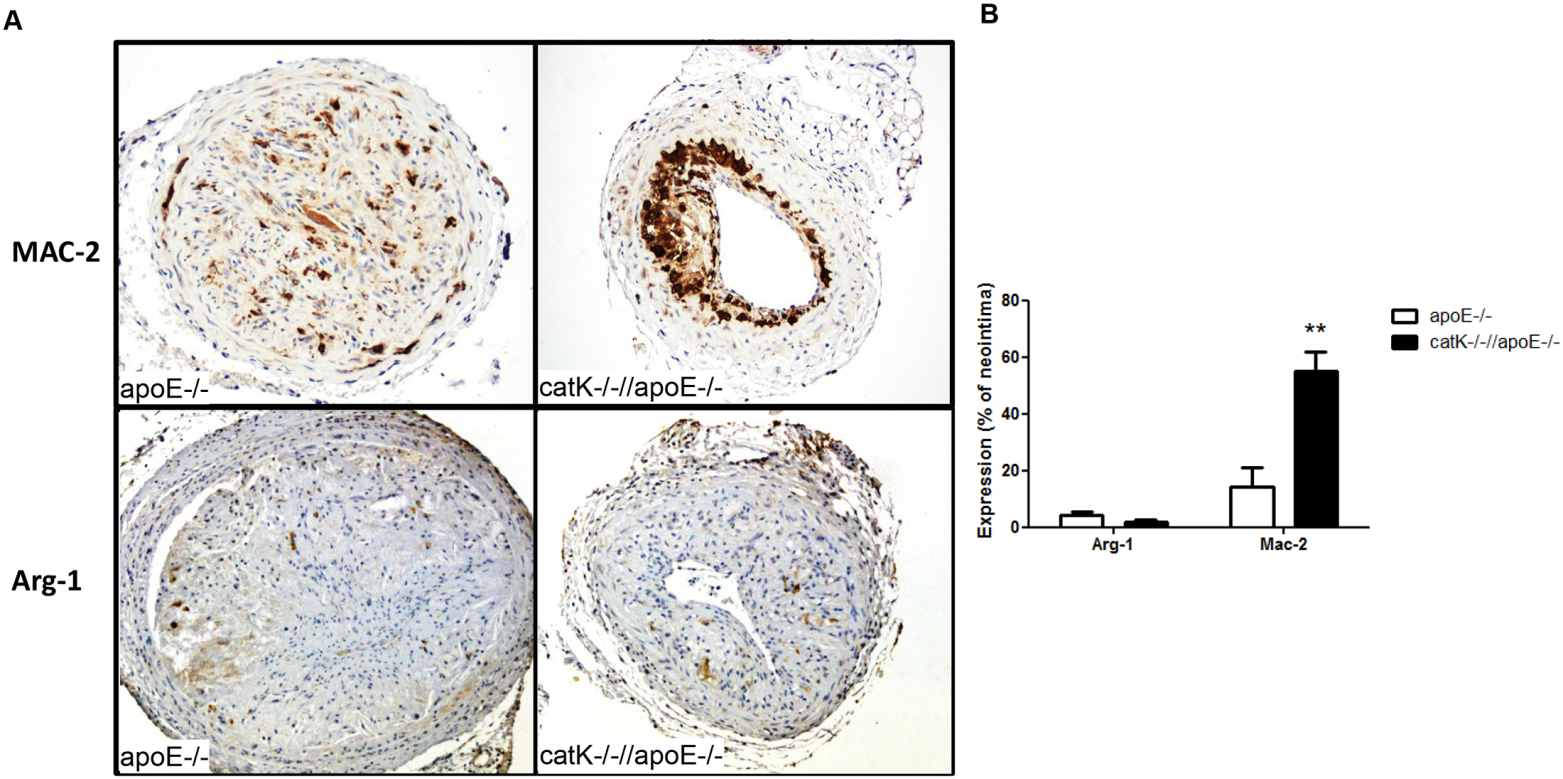
CatK defiency increases expression of the M2a marker Mac-2/Galectin-3 in ApoE^-/-^ mice. (A) Representative pictures of Mac-2 (upper panels) and Arg-1 (lower panels) staining in apoE^-/-^ and catK^-/-^//apoE^-/-^ mice. (B) While Arg-1 positive cells were scarce and not different between groups, CatK deficiency increased Mac-2 positive cells in the intima of ApoE^-/-^ mice. The number of Mac-2 and Arg-1 positive cells in the neointima were corrected for corresponding lesion area or total cells, respectively. Values represent the mean ± SEM. (n = 4–6 for each group).

In vivo, we analyzed expression of the M2a markers Arg-1 and Mac-2/Gal-3[16] in lesions of catK^-/-^//apoE^-/-^ and apoE^-/-^ mice. Although Arg-1+ cells were relatively scarce and not significantly different between groups, at least at this time point (i.e. 4 weeks after ligation), we found a marked 3.9-fold upregulation of Mac-2 in lesions of CatK deficient mice (p = 0.003, n = 4 vs 6, Fig 6). Enhanced M2a macrophage polarization, which is crucially involved in wound healing responses, in catK deficient mice might explain the reduced outward remodeling response in macrophage-rich neointima formation in the apoE^-/-^ background.

## Discussion

Vascular remodeling is defined as the adaptation of vascular wall geometry in response to (patho)physiologic stimuli [17]. It includes a progressive and persistent change in vessel size and structural alterations of the vessel wall, including degradation and reorganization of the ECM. Vascular remodeling is a common feature both in atherosclerosis, after invasive vascular interventions, such as percutaneous transluminal coronary angioplasty, and in flow cessation- induced vessel retraction [6, 7, 9, 17]. The key role of the cystein protease catK in atherosclerosis [5] and proteolysis of ECM and in particular collagen [2, 18] led us to investigate the contribution of this protease in flow cessation-induced remodeling of the carotid artery in both macrophage-poor and macrophage-rich lesions of C57Bl6 wt and apoE^-/-^ mice, respectively. We here show that catK deficiency normalizes the excessive, inflammatory intimal hyperplasia observed in hyperlipidemic apoE^-/-^ mice, suggesting that catK is a key player in apoE^-/-^ associated vascular remodeling in flow cessation. Moreover, catK may do so by modulating monocyte differentiation and in particular by modulating macrophage polarization rather than via its elastolytic activity. Indeed, SMC-driven vascular remodeling in normolipidemic C57Bl6 mice was not affected by catK deficiency.

Pivotal in vascular remodeling are degradation of the elastic laminae and the ECM, vascular inflammation and SMC phenotypic changes. The apoE^-/-^ mouse has been used to study vascular remodeling processes under pathophysiological conditions, and ligation of the carotid artery in these mice was employed to emulate flow cessation-induced vascular remodeling and inflammatory hyperplasia [9, 19, 20]. In this model, remodeling processes are dependent on both macrophage content [9] and macrophage-derived mediators, such as proteases [21] Outward remodeling was postulated to be driven by macrophage expression of matrix metalloproteinase (MMP), which was supported by the observation that MMP9^-/-^//apoE^-/-^ mice showed a prominent reduction in the total vessel and intima area after carotid artery ligation [21]. In contrast to the study by Lessner et al, in which MMP9 deficiency resulted in a decrease in collagen and SMC content, we did not observe any changes in the relative collagen and SMC content in lesions of catK deficient apoE^-/-^ and wt mice, despite a complete normalization of the aggravated remodeling response in the former. As discussed above, our study also did not reveal any effect of catK deficiency on elastin laminae fragmentation in both mouse strains. Apparently the reduced proteolytic activity in catK deficiency may only partly underlie the normalized vascular remodeling response to carotid artery ligation in apoE^-/-^ mice.

Interestingly, in addition to its role in ECM degradation, catK was also reported to have pro-inflammatory activity by modulating inflammatory signaling pathways such as TGF-β [3] and TLR-9 [4]. Therefore, we investigated the inflammatory status of catK deficient mice in more detail. ApoE^-/-^ mice showed increased levels of circulating NK cells and decreased Treg cell numbers compared with wt mice. Of note, catK deficiency in apoE^-/-^ mice was accompanied by dramatically reduced systemic CD3+ and NK cell counts and an increase in Treg cell population, which in fact were normalized to base levels of wt mice, whereas no effects on these cell populations were seen in the wt mice. Although evidence implicating the direct role of regulatory T cell on vascular remodeling is lacking, regulatory T cell deletion significantly augmented post-ischemic neovascularization [22]. These effects were correlated with enhanced accumulation of CD3+ T cells in the ischemic leg [22]. On the other hand, treatment of CD28^–/–^mice with anti-CD3+ antibody enhanced the number of endogenous Treg cells and led to a significant reduction of the postischemic inflammatory response and neovascularization [22]. Thus, regulatory T cell might inhibit outward remodeling through abrogating inflammatory responses.

CatK deficiency did not only alter T-cell but also myeloid differentiation. Phenotypically, Ly6C^high^ subsets of monocytes are characterized as inflammatory monocytes. Ly6C^high^ monocytes have the capacity to migrate to sites of peripheral inflammation and are important for the resolution of inflammation, whereas Ly6C^-^ monocytes enter the tissues and replenish the tissue-resident macrophage and DC population [23]. Surprisingly, apoE^-/-^ mice showed significantly decreased level of circulating CD11b+Ly6G-Ly6C^high^ monocytes compared with wt mice. This finding is not consistent with the previous report showing an elevated Ly6C^high^monocyte in circulation of apoE^-/-^ vs wt mice [24]. Interestingly, the blood monocyte population of catK^-/-^//apoE^-/-^ mice was enriched in inflammatory CD11b+Ly6G-Ly6C^high^, whereas that of resident Ly6C^-^ monocytes was significantly reduced compared with apoE^-/-^ mice. Thus, catK deficiency under apoE^-/-^ background normalized Ly6C^high^ monocyte population to base level of wt background. The implications of this finding for the normalized vascular remodeling in catK deficiency remain to be addressed.

CatK deficiency may not only affect monocyte differentiation but also monocyte/macrophage polarization. Our data show that catK deficiency enhances Arg-1 expression in bone-marrow derived macrophages, indicating a shift of apoE^-/-^ BMM to a wound healing macrophage phenotype [25, 26]. Arginase-1 is a pro-fibrotic enzyme that shifts the arginine metabolism towards the production of proline and polyamines, and a well-known marker of M2a macrophages [25]. In contrast to the bone marrow-derived macrophages, Arg-1 expression in neointima was not different between groups, but expression of arginase in general was relatively low. This is in line with an observation by Ostriker et al. showing that in presence of SMC-conditioned medium arginase expression in macrophages was significantly reduced[27]. Nevertheless, macrophages in apoE^-/-^ neointimal lesions did display a significantly upregulated protein expression of Mac-2/Galectin-3 in catK^-/-^//apoE^-/-^ mice. Gal-3 has been reported as marker of alternatively activated M2a macrophages [16] and is known to regulate cytokine expression, efferocytosis capacity as well as adhesion, migration and apoptosis of macrophages[28]. Interestingly, both arginase-1 and Gal-3 have been shown to be regulated by TGF-β signaling [29]. Previously, we showed catK deficiency upregulated TGF-β expression, which could subsequently lead to the observed enhanced arginase-1 and gal-3 expression and concomitant M2a polarization. The inhibitory effects of catK deficiency on intimal hyperplasia in apoE^-/-^ mice could at least be partly attributable to a shifted macrophage polarization. The molecular mechanism underlying the anti-inflammatory potential of catK deficiency might involve TLR9 and TGF-β signaling pathway. CatK inhibition led to reduced TLR9 responses [4] and stimulated TGF-β signaling [3]. Although TLR4 was reported to be involved in outward arterial remodeling in atherosclerotic apoE3 Leiden transgenic mice [30], the role of TLR9 in vascular remodeling is less defined. In this study, we focused on the role of catK deficiency on inflammatory status. Nevertheless, whether or not catK deficiency will influence lesion component of T cells, macrophage polarization or specifically TLR9 and TGF-β activity remains to be addressed.

In summary, catK deficiency completely normalized the increased vascular remodeling response to flow cessation in apoE^-/-^ mice, but did not affect macrophage-poor lesion formation in wild type mice. This identifies catK as a major culprit in flow cessation-induced inflammatory remodeling. Furthermore our data suggest that catK deficiency does so by inhibiting the apoE^-/-^ associated hyperinflammatory status, rather than by attenuating ECM degradation.

## Supporting Information

S1 Fig The absence of catK did not affect serum total cholesterol in both wt and apoE^-/-^ mice. Values represent the mean ± SEM. (n = 14–15 for apoE^-/-^ and catK^-/-^//apoE—^/-^ mice; n = 7 for wt and catK^-/-^ mice).
(PDF)

S2 Fig Quantification of collagen types in wt and CatK-/- neointima, expressed as percentage of neointimal area. Colours indicate various collagen structures (ranging from loosely patched, immature, thin collagen (green) to tightly packed, mature, thick collagen fibers (red)).
(PDF)

S1 Table Primer Sequences. (PDF)
