# Supplementary figures and images for: Cathepsin K Deficiency Prevents the Aggravated Vascular Remodeling Response to Flow Cessation in ApoE-/- Mice
Source: PLoS One. 2016 Sep 16;11(9):e0162595. doi: 10.1371/journal.pone.0162595 (PMC5026377; doi:10.1371/journal.pone.0162595)

S1 Fig.

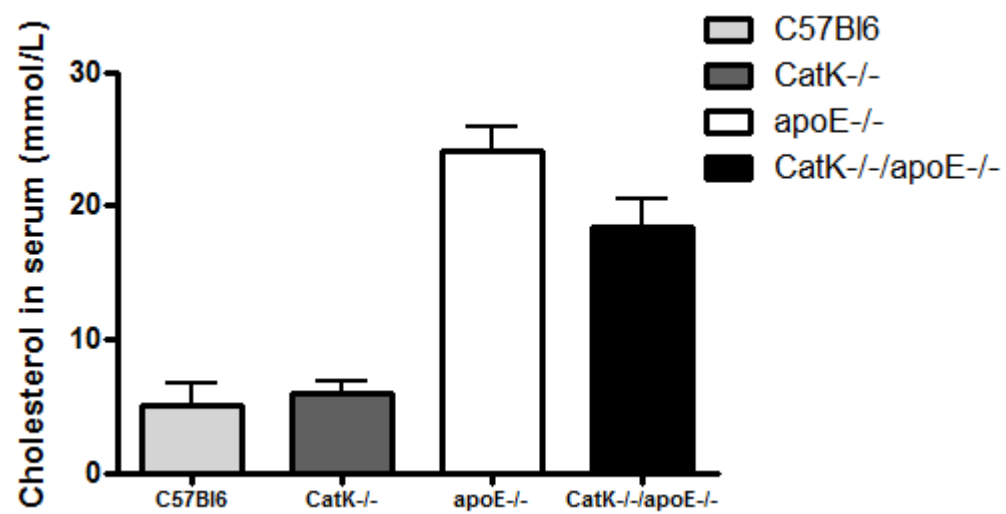

Supplement: S1 Fig — Values represent the mean ± SEM. (n = 14–15 for apoE-/- and catK-/-//apoE—/- mice; n = 7 for wt and catK-/- mice). (PDF) [file pone.0162595.s001.pdf]

S2 Fig.

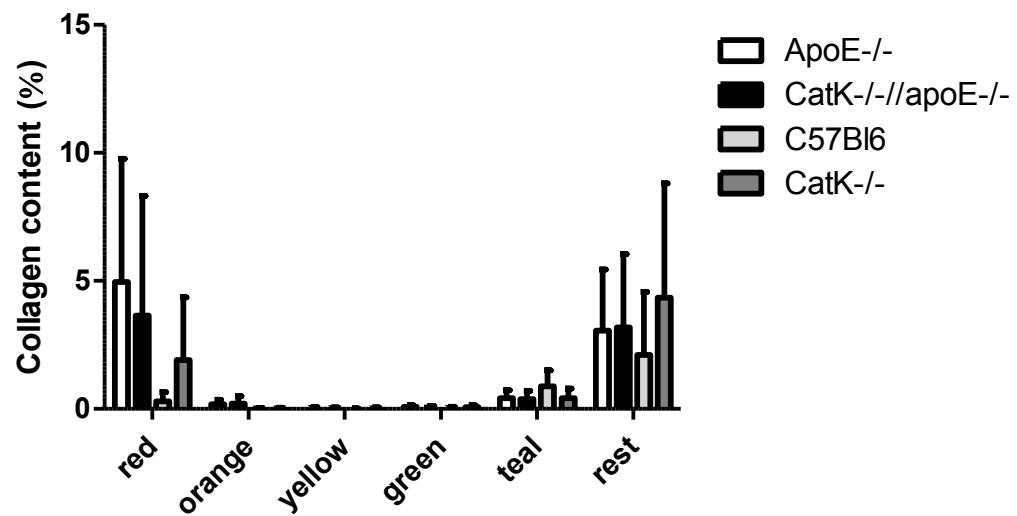

Supplement: S2 Fig — Colours indicate various collagen structures (ranging from loosely patched, immature, thin collagen (green) to tightly packed, mature, thick collagen fibers (red)). (PDF) [file pone.0162595.s002.pdf]
