# Supplementary material for: Cathepsin K Deficiency Prevents the Aggravated Vascular Remodeling Response to Flow Cessation in ApoE-/- Mice
Source: PLoS One. 2016 Sep 16;11(9):e0162595. doi: 10.1371/journal.pone.0162595 (PMC5026377; doi:10.1371/journal.pone.0162595)
Supplement: S1 Table — (PDF) [file pone.0162595.s003.pdf]

S1 Table

	Forward primer (5’-3’)	Reverse primer (5’-3’)
**Arginase-1**	ATGGAAGAGACCTTCAGCTAC	GCTGTCTTCCCAAGAGTTGGG
**MR**	GCAAATGGAGCCGTCTGTGC	CTCGTGGATCTCCGTGACAC
**IL-10**	TCTTACTGACTGGCATGAGGATCA	GTCCGCAGCTCTAGGAGCAT
**Nos2**	CAGCTGGGCTGTACAAACCTT	CATTGCAAGTGAAGCGTTTCG
**IL-18**	ACAACTTTGGCCGACTTCAC	GGGTTCACTGGCACTTTGAT
**cyclophilin**	CAAATGCTGGACCAAACACAA	TTCACCTTCCCAAAGACCACAT
